# Training family physicians and residents in family medicine in shared decision making to improve clinical decisions regarding the use of antibiotics for acute respiratory infections: protocol for a clustered randomized controlled trial

**DOI:** 10.1186/1471-2296-12-3

**Published:** 2011-01-26

**Authors:** France Légaré, Michel Labrecque, Gaston Godin, Annie LeBlanc, Claudine Laurier, Jeremy Grimshaw, Josette Castel, Isabelle Tremblay, Pierre Frémont, Michel Cauchon, Kathleen Lemieux, Caroline Rhéaume

**Affiliations:** 1 Research Center of Centre Hospitalier Universitaire de Québec, Hospital St-François D'Assise, Knowledge Transfer and Health Technology Assessment Research Group, 10 Espinay, Québec, QC, G1L 3L5, Canada; 2 Department of Family Medicine and Emergency Medicine, Faculty of Medicine, Université Laval, PavillonVandry, Cité Universitaire, Québec, QC, G1K 7P4, Canada; 3 Faculty of Nursing, Université Laval, PavillonVandry, Cité Universitaire, Québec, QC, G1K 7P4, Canada; 4 Knowledge and Encounter Unit, Mayo Clinic, 200 First Street SW, Rochester, MN, 55905, USA; 5 Faculty of Pharmacy, Université de Montréal, Pavillon Jean-Coutu, Montréal, QC, H3T 1J4, Canada; 6 Clinical Epidemiology Program, Ottawa Hospital Research Institute, Civic Campus, Ottawa, ON, K1Y 4E9, Canada

## Abstract

**Background:**

To explore ways to reduce the overuse of antibiotics for acute respiratory infections (ARIs), we conducted a pilot clustered randomized controlled trial (RCT) to evaluate DECISION+, a training program in shared decision making (SDM) for family physicians (FPs). This pilot project demonstrated the feasibility of conducting a large clustered RCT and showed that DECISION+ reduced the proportion of patients who decided to use antibiotics immediately after consulting their physician. Consequently, the objective of this study is to evaluate, in patients consulting for ARIs, if exposure of physicians to a modified version of DECISION+, DECISION+2, would reduce the proportion of patients who decide to use antibiotics immediately after consulting their physician.

**Methods/design:**

The study is a multi-center, two-arm, parallel clustered RCT. The 12 family practice teaching units (FPTUs) in the network of the Department of Family Medicine and Emergency Medicine of Université Laval will be randomized to a DECISION+2 intervention group (experimental group) or to a no-intervention control group. These FPTUs will recruit patients consulting family physicians and residents in family medicine enrolled in the study. There will be two data collection periods: pre-intervention (baseline) including 175 patients with ARIs in each study arm, and post-intervention including 175 patients with ARIs in each study arm (total n = 700). The primary outcome will be the proportion of patients reporting a decision to use antibiotics immediately after consulting their physician. Secondary outcome measures include: 1) physicians and patients' decisional conflict; 2) the agreement between the parties' decisional conflict scores; and 3) perception of patients and physicians that SDM occurred. Also in patients, at 2 weeks follow-up, adherence to the decision, consultation for the same reason, decisional regret, and quality of life will be assessed. Finally, in both patients and physicians, intention to engage in SDM in future clinical encounters will be assessed. Intention-to-treat analyses will be applied and account for the nested design of the trial will be taken into consideration.

**Discussion:**

DECISION+2 has the potential to reduce antibiotics use for ARIs by priming physicians and patients to share decisional process and empowering patients to make informed, value-based decisions.

**Trial Registration:**

ClinicalTrials.gov: NCT01116076

## Background

The use of antibiotics for acute respiratory infections (ARIs) has been likened to the tragedy of the commons--an event where individuals who acted locally for their own benefit inadvertently contributed to catastrophe at the ecological level [1]. ARIs are the main reported reason why patients consult primary care physicians in North America [2]. In a study that assessed the accuracy of primary care physicians' billing claims in the province of Quebec, Canada, 1,173 of 3,526 visits (33.3%) produced a diagnosis of ARI later confirmed by chart audit [3]. Yet the rate of use of antibiotics for ARIs is well above the expected prevalence of bacterial infection. For example, in a study based on` the National Research System of the College of Family Physicians of Canada, of 408 clinical encounters for respiratory tract infections (52.6% for acute bronchitis, 23.3% for an undiagnosed condition and 15.5% for viral illness), in 56.9% of visits, the patient received antibiotics immediately [4]. This figure contrasts with the study's observation that in 70% of the encounters, family physicians (FPs) expressed uncertainty about the need for antibiotics [4]. A second study, this one of Ontario children under 16, recorded that of 4344 observed visits, 1706 resulted in a prescription for antibiotics [5]. Of these prescriptions, 1577 (92%) were for ARIs, of which 920 (53%) were for acute otitis media; a full course of antibiotics was given to 321 (76%) of 425 children with pharyngitis immediately [5]. Given that only 38% of adults with acute rhinosinusitis, 6 to 18% of children with an ARI, and 5 to 15% of adults with pharyngitis have a bacterial infection [6,7], this suggests that antibiotics are overused, a phenomenon that in the words of Patrick and Hutchison (2009) may build a population's resistance to antibiotics and reduce the success of future therapy [1].

To date, attempts to improve the clinical decision-making process regarding the use of antibiotics for ARIs have been moderately effective at best [8]. The clinical decision-making process is not always simple, as it requires the parties to reconcile different expertise: that of the health provider, considered a medical expert, and that of the patient, considered an expert in his/her personal values [9]. It is that context of dual knowledge that is of interest to research in shared decision making (SDM). Said to be the crux of patient-centred care [10], SDM is a process in which a healthcare choice is made by the clinician and the patient, working together [11]. In the context of deciding whether to use antibiotics for ARIs, the patient would participate in the decision by clarifying what s/he values most: maximizing the probability of recovery or minimizing the probability of side effects. SDM promotes decision making in the context of scientific uncertainty because it exposes the uncertainty inherent to the clinical decision-making process by requiring the parties to discuss benefits and risks [12]. In others words, SDM is about improving the patient-clinician decision-making process so that decisions lead to a choice that is not only informed by the best evidence but is also in line with what patients most value. We argue that SDM has the potential to improve the clinical decision-making process regarding antibiotic use for ARIs by empowering patients (again through enhanced communication skills and prognostic understanding) to make informed decisions about when to take further action [13,14].

In 2008, we completed a pilot clustered randomized controlled trial (RCT) studying antibiotics prescriptions for ARIs in primary care [15]. We randomized four family medicine groups (FMGs) to an intervention group (two FMGs, 18 FPs, and 173 patients) or to a control group (two FMGs, 15 FPs, and 147 patients). We found that FPs' exposure to DECISION**+**, a multifaceted intervention for implementing SDM in medical practices that included training, reminders and feedback, was associated with a decrease in decisions for "immediate antibiotics" compared to decisions for "delayed/no antibiotics" (49% vs. 33% absolute difference = 16%; p = 0.08 and relative risk = 0.69; 95% CI: 0.47-1.03). By "immediate antibiotics," we refer to the patient's decision, made during the consultation, to use antibiotics immediately to treat the ARI. Associated with the main outcomes of this pilot trial that demonstrated the feasibility and acceptability [16], we are now conducting a large clustered RCT of a modified version of DECISION+, DECISION+2. The proposed trial will be the first RCT with sufficient statistical power to assess the impact of DECISION+2 on ARI patients reporting a decision to use antibiotics immediately. This information will inform strategies that cause the parties to work together to improve the clinical decision-making process for the treatment of ARIs. In so doing, the RCT will address the problem of antibiotic overuse for ARIs in primary care and eventually improve population health.

The proposed RCT is based on our conceptual framework [17] and the results of the pilot RCT [15,16]. Its first objective is to evaluate how DECISION+2 impacts the proportion of ARI patients reporting a decision to use antibiotics immediately. The other objectives are to estimate the impact of DECISION+2 on 1) the decisional conflict scores of physicians and their patients and the level of agreement between their scores, 2) perception of patients and physicians that SDM occurred, 3) patients' adherence to the decision made, 4) consultation for the same reason, 5) patients' decisional regret, 6) patients' quality of life, and 7) physicians and patients' intention to engage in SDM in future clinical encounters dealing with antibiotics for ARIs.

## Methods/Design

### Study design

The study is a multi-center, two-arm, parallel clustered RCT conducted in the network of the 12 family practice teaching units (FPTUs) from the Department of Family and Emergency Medicine at Université Laval. All FPTUs that will accept to participate will be randomly allocated to either 1) an experimental group that is exposed to the DECISION+2 intervention or 2) a control group with no intervention. Two data collection periods are planned for each study group: the first before the DECISION+2 intervention is offered and the second after the post-DECISION+2 intervention.

### Study population

#### Family Practice Teaching Units and their Family Physicians

Following the approval by Family Medicine residency program committee at Université Laval, the 12 FPTU directors will be contacted by means of a letter sent by the research team briefly explaining that a new SDM training course addressing the use of antibiotics in the context of ARIs will be formally evaluated by means of a RCT, and that the participation of their FPTU to the trial would be appreciated. Meetings with all health professionals in interested FPTU will be organized to further explain implications of participating to the trial.

All FPs, both teachers and residents, who provide care in the FPTU's walk-in clinics, will be eligible to participate in the trial. FPs will be excluded from the study if they were involved or had participated in the DECISION+ pilot RCT, or if they do not expect to practice at the FPTU for the study duration (e.g., residents ending their residency program, physicians doing rotations outside of the FPTU, physicians who expect to be pregnant, physicians planning to retire).

Should the FPTU agree to participate, the FPTU director will sign a letter to this effect. The letter will be returned to the research institution's ethics committee. All participants will sign an informed consent form approved by the research institution's ethics committee.

#### Patients

Patients will be included if 1) they are ≥ 18 years old; if they are ≤17 years old but are accompanied by a parent or legal guardian; 2) they are consulting a participating physician for an ARI (e.g., acute otitis media, acute rhinosinusitis, acute pharyngitis, or acute bronchitis) for which an antibiotic treatment may be considered; 3) either the patient or the accompanying parent or guardian is able to read, understand and write French (expected level: Grade 8); and 4) they sign the informed consent form approved by the ethics committee of the Centre de Santé et des Services Sociaux de la Vieille-Capitale. This ethics committee approved this project on June 14^th ^2010. Patients with a condition requiring emergency care will not be eligible to participate.

### Interventions

#### Experimental group

In line with the Family Medicine residency program courses and following an in-depth evaluation with participants to the pilot trial [18], the original DECISION+ training program (i.e., 3 three-hour on-site interactive workshops, reminders and feedback) was modified to include a 2-hour web-based tutorial followed by a 2-hour on-site interactive workshop followed by reminders. The web-based tutorial addresses key components of the clinical decision-making process regarding antibiotic treatments for ARIs in primary care: 1) the probabilistic nature of a diagnosis of a bacterial versus a viral infection; 2) scientific evidence regarding the risk/benefit ratios of the options; 3) communication techniques; and 4) strategies to foster patients' participation in the decision-making process [17]. The interactive workshop aims to help physicians to review and integrate the concepts they acquired during the web-based training in such a way as to promote their patients' participation in SDM. Both the tutorial and the workshop include videos, exercises and decisional support tools to help FPs communicate to patients the probability of a bacterial ARI and the benefit/risk ratio associated with the use of antibiotics. Reminders will consist in the following strategy: during the second data collection period, research assistants will give participating physicians in the intervention group the decision support tools before recruiting ARI patients. In this large clustered RCT, the feedback component of the original DECISION+ training program will not be used.

Participants will have one month to complete the web-based self-tutorial, which lasts approximately 120 minutes. Collaborators have tested and appraised the web-based platform associated with the tutorial. After the tutorial completion period, the on-site interactive workshop will be offered to the experimental group. The on-site interactive workshop will be led by team members and facilitators trained during the DECISION+ pilot RCT. The facilitators of the workshop will be further trained during a standardization session prior to conducting the workshop in their FPTU. During the workshop participating FPs and residents will be instructed to use the material and decision support tools with their patients.

#### Control group

The FPs and the patients in the control group will not receive any particular intervention during the trial period. No specific course or training activity on SDM or on treatment of ARIs will be planned in the control FPTU over this period. In addition to avoid contamination bias, access to the web-based platform will be denied to the control group participants during the trial. The control group will be offered the experimental training after the completion of the proposed trial.

### Allocation of participants to trial groups

In this study, our unit of randomization is the FPTU. Randomization of all FTPU accepting to participate to the trial will be performed simultaneously by an experienced biostatistician who will use Internet-based software. Simultaneous randomization of all participating unit assures allocation concealment.

### Outcome measures

#### Primary outcome

The primary outcome will be the proportion of patients reporting a decision to use antibiotics immediately.

#### The decision to use antibiotics

After each clinical encounter for ARIs (e.g., acute otitis media, acute bronchitis, acute pharyngitis, or acute rhinosinusitis), patients and their FPs will indicate whether they discussed the use of antibiotic to treat the ARI and whether they decided to use antibiotics immediately, delay using antibiotics, or not use antibiotics. In the context of antibiotic overuse for ARI, limiting the decision for "immediate antibiotics" is the important target to achieve. Therefore, we will combine "delayed" and "no antibiotics" into a single category and contrast it with "immediate antibiotics."

We will validate patients' answers 1) by asking them to show their prescription to the research assistant, if applicable, who will note the medication prescribed and the date for filling the prescription, if given; and 2) by asking the same question to the FP after the index consultation. In our pilot RCT, agreement between FPs and patients was very high (Kappa = 0.90; p < 0.001) [15,16].

### Secondary outcomes

Secondary outcome measures assess the impact of DECISION+2 by evaluating: 1) physicians and patients' decisional conflict; 2) the agreement between the parties' decisional conflict scores; and 3) perception of patients and physicians that SDM occurred. In patients, we will also assess at 2 weeks: 1) adherence to the decision; 2) consultation for the same reason; 3) decisional regret; and 4) quality of life. Also, in both patients and FPs, we will assess their intention to engage in SDM in clinical encounters dealing with the use of antibiotics for ARIs in the future.

#### Decisional conflict

Decisional conflict will be assessed by administering the Decisional Conflict Scale after the clinical encounter. This questionnaire is similar for physicians and patients. It includes 19 items, each of which is scored on a 5-point Likert scale (1 = strongly agree to 5 = strongly disagree) where higher scores are associated with greater decisional conflict. Both the physician and the patient versions of the scale have adequate psychometric properties [15,19].

#### Perception of patients and physicians that SDM occurred

The perception of patients and FPs that SDM occurred will be assessed using a self-administered questionnaire, D-OPTION, derived from the original third observer instrument, OPTION [20,21].

#### Adherence to the decision

Two weeks after the clinical encounter, in a telephone interview, a research assistant will ask the patient two questions regarding their adherence to the decision: "Two weeks ago, what decision did you make with your FP about using antibiotics for your ARI?" (to use antibiotics immediately, delay using antibiotics, or not use antibiotics). and "Can you say that you maintained this decision?" (yes or no). If patient answers no, the research assistant will use an open-ended question to inquire about his/her reasons and determine what the patient did instead.

#### Consultation for the same reason

In line with the index consultation for which they were included in this trial, we will ask patients a simple question: "In the past two weeks, have you consulted a primary care provider for the same reason or for the same clinical problem?" (yes or no).

#### Decisional regret

During the same telephone call, the research assistant will assess the patient's decisional regret using the Decisional Regret Scale. The Decisional Regret Scale is a 5-item scale with efficient psychometric properties that correlates strongly with decision satisfaction and overall quality of life [22].

#### Quality of life

Patients’ quality of life will be assessed, immediately before and two weeks after the clinical encounter. In both cases, the research assistant will use the Short Form-12 questionnaire (SF-12V2 Health survey) [23] to measure the patient's general health status from his/her point of view. This questionnaire evaluates eight elements commonly held to represent health status: physical fitness, role functioning, pain, general health, vitality, social abilities, and emotional and mental health.

#### Intention to engage in SDM in future consultations dealing with antibiotics for ARIs

This intention on the part of FPs and their patients will be assessed by questionnaire in reference to the Theory of Planned Behaviour [24]. Composed of 15 items scored with a 7-point Likert scale, the questionnaire covers the theory's constructs, namely, attitudes, subjective norm, perceived behavioural control, and intention. Patients will complete this questionnaire before and 2 weeks after the clinical encounter; physicians will complete it once during the baseline data collection period and once again after the post-intervention data collection period. From a theoretical perspective, the questionnaire will ensure that we understand what underlying mechanisms should be considered when developing interventions to apply SDM in primary care.

### Other variables (potential effect modifiers)

#### Sociodemographic characteristics

Before the clinical encounter, patients will be assessed in terms of the following sociodemographic characteristics: age, gender, highest educational degree, employment, annual household income, size of household, marital status and health. FPs' characteristics will also be determined before the clinical encounter, during the first data collection period. We will assess FPs' age, number of years in practice, gender, number of formal years of education, other degrees, participation in committees, continuing professional development activities, and anxiety about uncertainty and disclosing uncertainty to patients [25,26].

### Data collection procedures

There will be two data collection periods: pre-intervention (baseline) and post-intervention. We will record data for FPs once during the baseline phase. Patients' characteristics will be recorded before their clinical encounter with the physician. After the clinical encounter, the patient and the physician will independently complete self-administered questionnaires that will assess both the decision that had been made (the primary outcome) and decisional conflict. Both the patient and the physician will also assess independently if SDM occurred. Patients will be contacted by phone two weeks after the clinical encounter to complete a short interview (adherence to the decision, consultation for the same reason, decisional regret, quality of life, and intention to engage in SDM). FPs' intention to engage in SDM regarding the use of antibiotics for ARIs will be recorded at baseline and again at the end of the study.

### Sample size and analysis

The primary outcome is whether or not patients report a decision for immediate use of antibiotics for ARI after an index consultation. In the pilot RCT, immediate use of antibiotics was 56% in the experimental group and 54% in the control group at baseline. We assume that the minimum clinical significance for an absolute reduction of immediate use of antibiotics is 20%. In order to detect a reduction from 60% to 40% with 80% power, at a 5% significance level, one would require a total of 194 patients in an individually randomized two-group trial. To take account of the clustering of participants within FPTU (unit of randomisation), we used an intracluster correlation coefficient (ICC) of 0.02 estimated from our pilot cluster RCT [15,16]. Therefore, we will need 288 patients to have the same power as an individually randomized trial, that is, 24 patients per cluster assuming that all 12 FPTUs (clusters) will participate. In order to compensate for loss due to follow up and missing data (as observed in our pilot RCT), we will recruit 350 patients at each data collection period, 175 patients per group per period.

We will perform descriptive statistical analysis of socio-demographic characteristics in order to assure the comparability of both groups (intervention and control). Potentially confounding variables will be introduced as covariates in statistical modelling analyses. Multilevel modelling will be used in order to take the hierarchical structure of the data into account by specifying random effects at each of the three levels: FTPU, FP and patient. For each outcome analysed, according to the type of variables (continuous or categorical), the goodness of fit and the assumptions of each model will be assessed. Statistical analysis will be performed using the SAS statistical package (SAS Institute Inc. 2005. SAS OnlineDoc^® ^9.1.3. Cary, NC: SAS Institute Inc.) and the MLM software MLwiN 2.10 Beta.

## Discussion

This RCT will be the first study with enough statistical power to assess the impact of DECISION+2 on reporting a decision for the immediate use of antibiotics for ARIs in the context of FPTUs. We expect DECISION+2 to foster SDM in consultations between FPs and their patients. The innovative approach proposed in this trial recognizes that translating and exchanging knowledge and implementing evidence in clinical practice result from a complex interplay of interpersonal elements that influence the clinical encounter. By revealing as yet unknown mechanisms that underlie knowledge transfer in the clinical context, DECISION+2 has the potential to foster new knowledge transfer strategies that optimize drug prescriptions.

## Competing interests

The authors declare that they have no competing interests.

## Authors' contributions

FL, ML, GG, AL, CL and JG collectively drafted the study protocol and sought funding and ethical approving. FL and ML are responsible for the management of the trial. All authors approved the final manuscript. FL is its guarantor.

## Pre-publication history

The pre-publication history for this paper can be accessed here:

http://www.biomedcentral.com/1471-2296/12/3/prepub
